# Isolation and characterization of *LEAFY*-homologous genes from two *Tricyrtis* spp. showing different inflorescence architecture

**DOI:** 10.5511/plantbiotechnology.25.0225a

**Published:** 2025-06-25

**Authors:** Sota Takanashi, Yuto Imamura, Haruki Ouchi, Shoichi Sato, Masahiro Otani, Masaru Nakano

**Affiliations:** 1 Graduate School of Science and Technology, Niigata University; 2 Faculty of Agriculture, Niigata University

**Keywords:** expression pattern, functional analysis, inflorescence architecture, *LEAFY*, *Tricyrtis* spp.

## Abstract

For ornamental plants, inflorescence architecture is one of the most important traits to determine their commercial values. However, molecular mechanisms of inflorescence architecture determination have not yet been fully elucidated. *LEAFY* (*LFY*), which encodes a plant-specific transcriptional factor, has been shown to play a key role in the switch from vegetative to reproductive phases. Recent studies have demonstrated that LFY is involved not only in floral meristem induction but also in inflorescence architecture determination. *Tricyrtis* spp., which belong to the family Liliaceae, show two different types of inflorescence architecture: *T*. *hirta* produces both apical and lateral flowers, whereas *T*. *formosana* produces only apical flowers. In the present study, we isolated *LFY*-homologous genes from *T*. *hirta* and *T*. *formosana* (designated as *ThirLFY* and *TforLFY*, respectively) and analyze their functions and expression patterns as a first step toward elucidation of molecular mechanisms of inflorescence architecture determination in *Tricyrtis* spp. Alignment analysis based on amino acid sequences showed that both ThirLFY and TforLFY have functional motifs of LFY, and only three amino acid differences are found between them. Transgenic *Arabidopsis thaliana* plants overexpressing *ThirLFY* or *TforLFY* showed early flowering and production of secondary inflorescences, and no functional differences were observed between ThirLFY and TforLFY. *In situ* hybridization analysis showed that *ThirLFY* was expressed in both apical and lateral buds of *T*. *hirta*, whereas *TforLFY* was only expressed in apical buds of *T*. *formosana*. Thus, two different types of inflorescence architecture in *Tricyrtis* spp. may be caused by different expression patterns of *LFY*-homologous genes.

## Introduction

*LFY*, which encodes a plant-specific transcriptional factor, has been shown to play a key role in the switch from vegetative to reproductive phases (Blazquez et al. 1997; Liljegren et al. 1999; Weigel et al. 1992). In *Arabidopsis thaliana*, *lfy* mutants exhibited late flowering and produced leaf-like abnormal tepals (Shannon and Meeks-Wagner 1993; Weigel et al. 1992). On the other hand, overexpression of *LFY* induced early flowering and production of terminal flowers (Weigel and Nilsson 1995).

Several studies have reported that *LFY* is also involved in determining inflorescence architecture. In *A*. *thaliana*, *lfy* mutants showed abnormal branching patterns through inflorescence-to-vegetative shoot conversion (Schultz and Haughn 1991; Weigel et al. 1992). Similar alterations in inflorescence architecture have also been observed in *lfy* mutants of *Antirrhinum majus*, *Solanum lycopersicum* and *Petunia hybrida* (Immink et al. 1999; Masiero et al. 2004; Molinero-Rosales et al. 1999). In addition, expression patterns of *LFY*-homologous genes in meristems have been reported to be different among four brassicaceous species showing different inflorescence architectures (Shu et al. 2000; Sliwinski et al. 2006, 2007; Yoon and Baum 2004). However, no direct evidence for the relationship between *LFY*-homologous genes and inflorescence architecture has yet been obtained because no transformation system has been established in these brassicaceous species except for *A*. *thaliana*.

*Tricyrtis* spp., which belong to the family Liliaceae, show two different types of inflorescence architecture: *T*. *hirta* produces both apical and lateral flowers, whereas *T*. *formosana* produces only apical flowers. In addition, an efficient *Agrobacterium*-mediated transformation system have already been established for *Tricyrtis* spp. (Adachi et al. 2005). Therefore, *T*. *hirta* and *T*. *formosana* are expected to be useful as model plants for molecular studies on inflorescence architecture. In the present study, we isolated and characterized *LFY*-homologous genes from these two species as a first step toward elucidation of molecular mechanisms of inflorescence architecture determination in *Tricyrtis* spp.

## Materials and methods

### Plant materials

Potted plants of *T*. *hirta* and *T*. *formosana* ‘Seiryu’ were cultivated in a greenhouse without heating. *A*. *thaliana* plants were cultivated in an incubator under a long-day conditions (16 h light/8 h dark) at 22°C.

### Isolation of *LFY*-homologous genes

Total RNAs were extracted from immature flower buds (ca. 5 mm in length) of potted *Tricyrtis* plants using RNeasy Plant Mini Kit (QIAGEN, Hilden, Germany) and treated with RNase-Free DNase (QIAGEN) according to the manufacturer’s instructions. In order to amplify full-length cDNA fragments of *LFY*-homologous genes, RACE was performed using 3′/5′-Full RACE Core Set (Takara, Shiga, Japan) according to the manufacturer’s instructions. Primers used for isolation of *LFY* homologous genes are listed in Supplementary Table S1.

### Phylogenetic analysis

Deduced amino acid sequences of *LFY*-homologous genes were aligned using ClustalW program (Thompson et al. 2002). Molecular phylogenetic tree based on amino acid sequences was constructed using the maximum likelihood method with 1,000 bootstrap replicates in MEGA-X (Felsenstein 1985; Kumar et al. 2018). Accession numbers of *LFY*-homologous genes used for phylogenetic analysis are listed in Supplementary Table S2.

### Southern blot analysis

Total DNAs were extracted from young leaves of potted *Tricyrtis* plants the using CTAB method (Doyle 1990; Doyle and Doyle 1987). DNAs were then digested with *Eco*RI, *Kpn*I, *Hind*III, *Xba*I or *Bam*HI. Common sequences in 3′-terminal regions among *LFY*-homologous genes of *Tricyrtis* spp. (647 bp) were used as a probe. DNA probe was prepared using PCR DIG Probes Synthesis Kit (Roche, Basel, Switzerland). Hybridization was carried out under 42°C for 16 h with gentle shaking. Blocking, washing and detection were performed using DIG Nucleic Acid Detection Kit (Roche) according to the manufacturer’s instruction.

### Transformation of *A. thaliana*

*Agrobacterium tumefaciens* strains, EHA101/pIG-*ThirLFY* and EHA101/pIG-*TforLFY*, were used. T-DNA regions of the binary vectors pIG-*ThirLFY* and pIG-*TforLFY* contain *LFY*-homologous gene of *T*. *hirta* and *T*. *formosana* respectively, both under the control of the CaMV35S promoter. T-DNA regions of both binary vectors also contain the NPTII gene under the control of the NOS promoter, and the HPT gene under the control of the CaMV35S promoter. *A*. *tumefaciens* strain EHA101/pIG-*AtLFY* (Sankhuan et al. 2022) was also used as a control. Transformation of *A*. *thaliana* was performed using the floral dip method (Clough and Bent 1998). Transgenic plants were cultivated as described above.

### Expression analysis by RT-PCR

Total RNAs were extracted from roots, stems, leaves and apical flower buds (ca. 5 mm in length) of potted *Tricyrtis* plants and then treated with RNase-Free DNase Set (QIAGEN) according to the manufacturer’s instructions. For cDNA synthesis, 500 ng of total RNAs were reverse-transcribed using PrimeScript™ RT reagent Kit (Takara) according to the manufacturer’s instructions.

RT-PCR was performed using EmeraldAmp® PCR Master Mix (Takara) on T100™ Thermal Cycler (Bio-Rad, California, USA). Each PCR reaction was carried out under the following conditions: denaturation at 94°C for 2 min; 30 cycles of 10 s at 98°C, 30 s at 58°C and 1 min at 72°C; and 72°C for 4 min. Amplified products were analyzed by electrophoresis on 1.5% agarose gels. The actin gene of *Tricyrtis* sp. *TrAct2* (accession number AB196260 in the GenBank/EMBL/DDBJ databases) was used as an internal standard. Primers used for RT-PCR analysis are listed in Supplementary Table S1.

### *In situ* hybridization

Apical and lateral buds were collected from potted *Tricyrtis* plants when apical flower buds had grown to ca. 5 mm in length. They were soaked in FAA (10% formalin, 5% acetic acid and 50% ethanol) with vacuum for 30–60 min and fixed for overnight at 4°C. Fixed samples were dehydrated in an increasing gradient series of ethanol and embedded in paraffin wax. Paraffin-embedded samples were cut into 10 µm sections using Microtome 2035 Biocut (Leica Biosystems, Nussloch, Germany).

RNA probe was designed for 647 bp of common sequences in 3′-terminal regions among *LFY*-homologous genes of *Tricyrtis* spp. and synthesized using DIG RNA Labelling Kit (Roche) according to the manufacturer’s instructions. Hybridization and detection were carried out following the DIG application manual for *in situ* hybridization (Roche).

## Results

### Isolation and phylogenetic analysis of *LFY*-homologous genes

**Figure 1:**
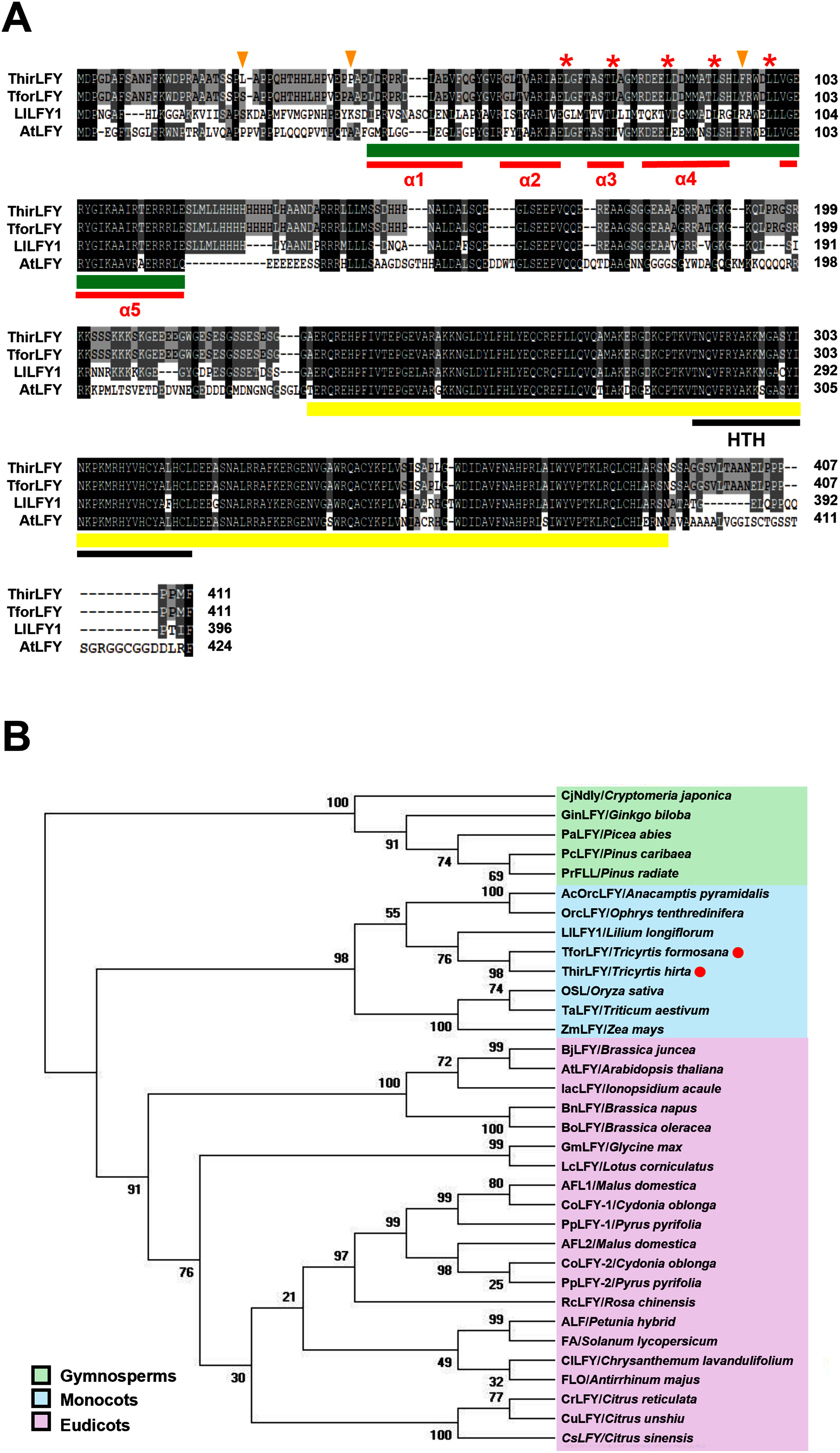
Figure 1. Alignment and phylogenetic analysis of ThirLFY and TforLFY based on the amino acid sequences. (A) Alignment of deduced amino acid sequences of ThirLFY (*Tricyrtis hirta*), TforLFY (*T*. *formosana*), LlLFY1 (*Lilium longiflorum*) and AtLFY (*Arabidopsis thaliana*). Green and yellow lines indicate conserved N- (include Sterile Alpha Motifs) and C-terminal regions (include DNA-binding domains) of LFY proteins, respectively. Red lines and asterisks indicate alpha helix structures and leucine residues, which are essential component for LFY protein conformation and homodimerization, respectively. Black lines indicate helix-turn-helix (HTH) structure, which is important motif for DNA-binding ability. Orange arrowheads indicate the positions of amino acid substitution between ThirLFY and TforLFY. (B) A molecular phylogenetic tree of *LFY*-homologous genes. Red spots indicate *ThirLFY* and *TforLFY*.

In order to identify full-length coding sequences of *LFY*-homologous genes of *T*. *hirta* and *T*. *formosana*, we performed RACE using cDNAs, which were synthesized from total RNAs extracted from immature flower buds (ca. 5 mm in length). The specific primers used for 3′-RACE were designed based on the nucleotide sequences of highly conserved C-terminal regions among *LFY* homologous genes from other plant species. *LFY*-homologous genes of *T*. *hirta* and *T*. *formosana* were successfully isolated and designated as *ThirLFY* (accession number AB829895 in the GenBank/EMBL/DDBJ databases) and *TforLFY* (accession number AB829896 in the GenBank/EMBL/DDBJ databases), respectively. Both *ThirLFY* and *TforLFY* encode 411 amino acid residues, and characteristic motifs of *LFY*-homologous gene, such as short leucine zipper and DNA-binding domain, have been conserved in their amino acid sequences (Figure 1A). There were only three substitutions in full-length amino acid sequences between *ThirLFY* and *TforLFY*: Leu/Ser at position 25, Pro/Ala at position 40, and Phe/Tyr at position 95 (Figure 1A). Amino acid sequences of *ThirLFY* and *TforLFY* showed 49.3–67.3% homologies to those of *LFY*-homologous genes of other plant species. Phylogenetic analysis showed that both ThirLFY and TforLFY belong to the monocotyledon clade and clustered closely with LlLFY1 from *Lilium longiflorum* (Figure 1B).

**Figure 2:**
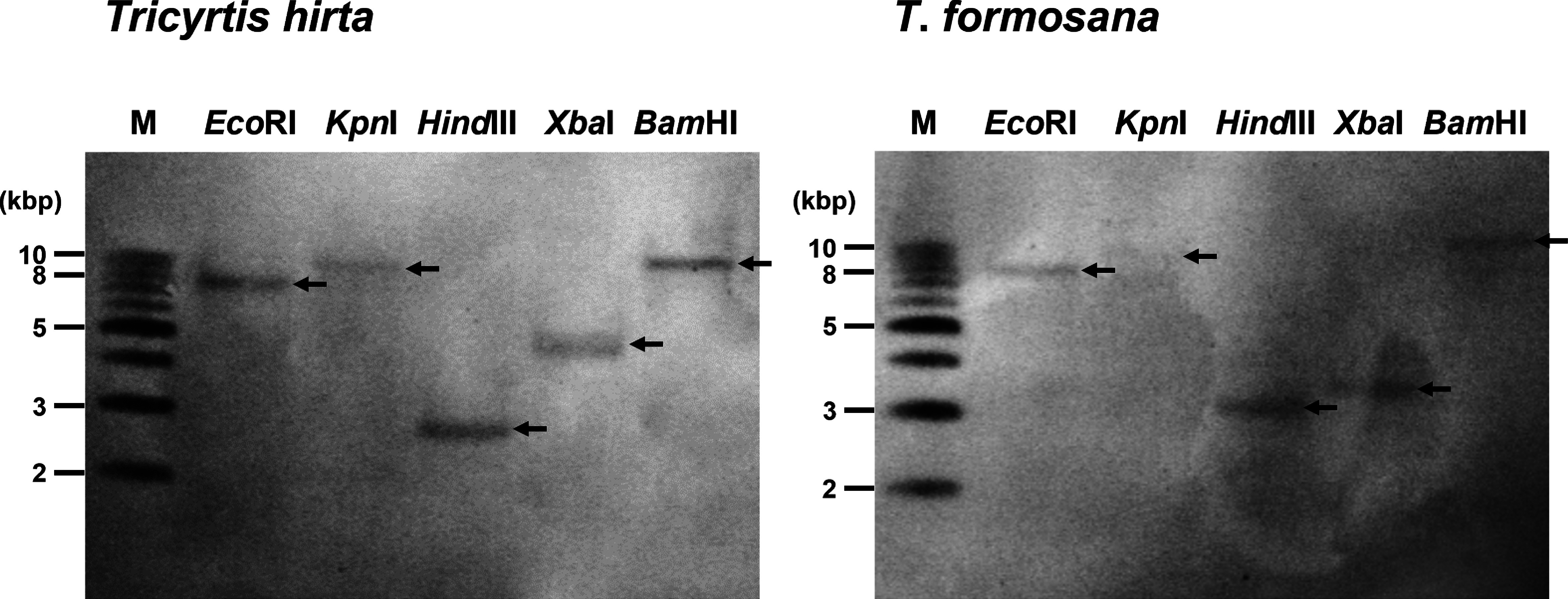
Figure 2. Southern blot analysis of *LFY*-homologous genes in the genome of *Tricyrtis hirta* and *T*. *formosana*. Genomic DNAs were digested with each of *Eco*RI, *Kpn*I, *Hind*III, *Xba*I and *Bam*HI.

In order to investigate the copy numbers of *LFY*-homologous genes in the genome of *T*. *hirta* and *T*. *formosana*, Southern blot analysis was performed. Only a single band was detected for all five restriction enzymes (*Eco*RI, *Kpn*I, *Hind*III, *Xba*I and *Bam*HI) in both *T*. *hirta* and *T*. *formosana*, indicating that both species have a single copy of *LFY*-homologous gene (Figure 2).

### Functional analysis of *LFY*-homologous genes

**Figure 3:**
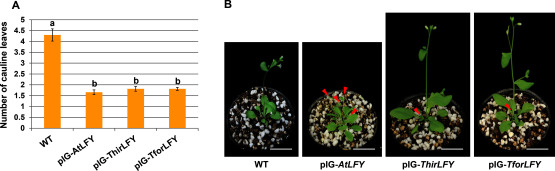
Figure 3. Transgenic *Arabidopsis thaliana* plants overexpressing *AtLFY*, *ThirLFY* and *TforLFY*. (A) Flowering time of wild-type and transgenic plants. Values represent the mean±standard error of 20, 8, 13, 17 biological replicates for the wild-type, pIG-*AtLFY*, pIG-*ThirLFY* and pIG-*TforLFY*, respectively. Mean values with different letters indicate significant difference at the 0.05 level with the Tukey–Kramer’s test. (B) Typical phenotypes of 30-day-old wild-type and transgenic plants. Red arrowheads indicate secondary inflorescence from axils of rosette leaves. Scale bars=2 cm.

In order to investigate the function of *ThirLFY* and *TforLFY*, we produced the overexpression constructs pIG-*ThirLFY* and pIG-*TforLFY*, and introduced them separately into *A*. *thaliana*. Totally 13 and 17 transgenic lines of *ThirLFY*- and *TforLFY*-overexpressing plants were obtained, respectively. Transgenic *A*. *thaliana* plants overexpressing *ThirLFY* or *TforLFY* exhibited early flowering compared with wild-type plants. The degree of early flowering in these transgenic plants was similar to that in transgenic plants overexpressing *AtLFY* (Figure 3A). Transgenic plants overexpressing *ThirLFY* or *TforLFY* produced secondary inflorescences from rosette leaf axils as in the case of those overexpressing *AtLFY* (Figure 3B).

### Expression analysis of *LFY*-homologous genes

**Figure 4:**
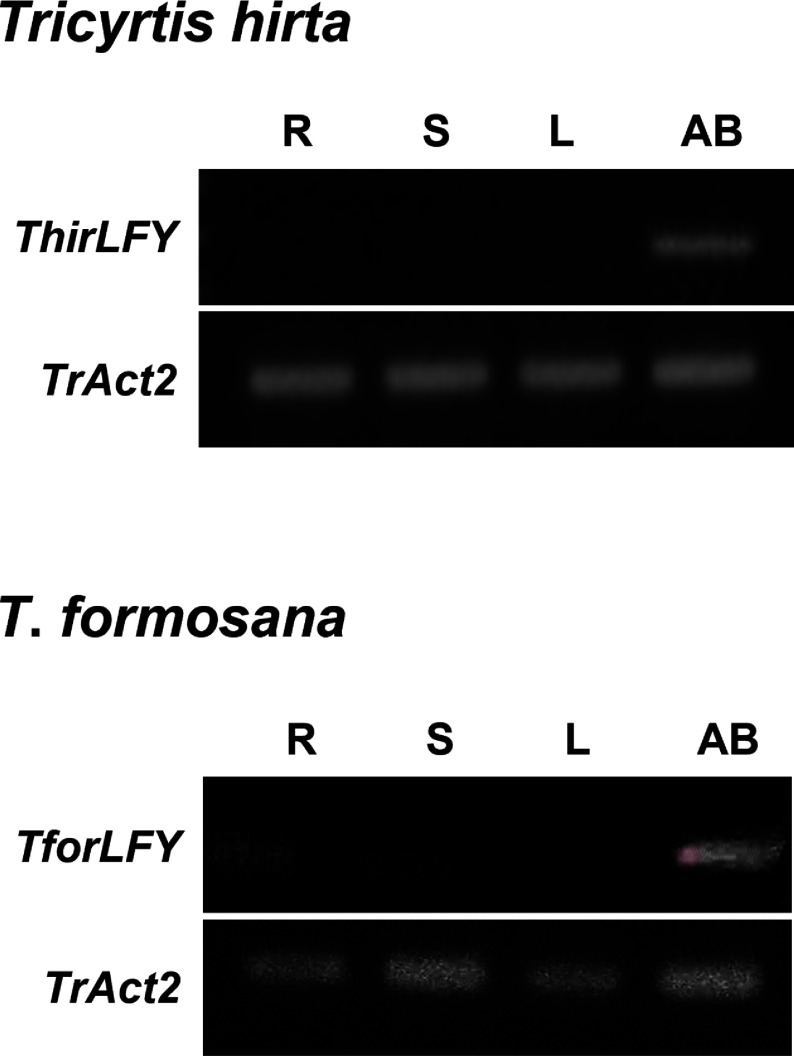
Figure 4. Semi-quantitative expression of *ThirLFY* and *TforLFY* in various tissues of *Tricyrtis hirta* and *T*. *formosana*. R, roots; S, stems; L, leaves; AB, apical flower buds. *TrAct2* was used as an internal standard.

**Figure 5:**
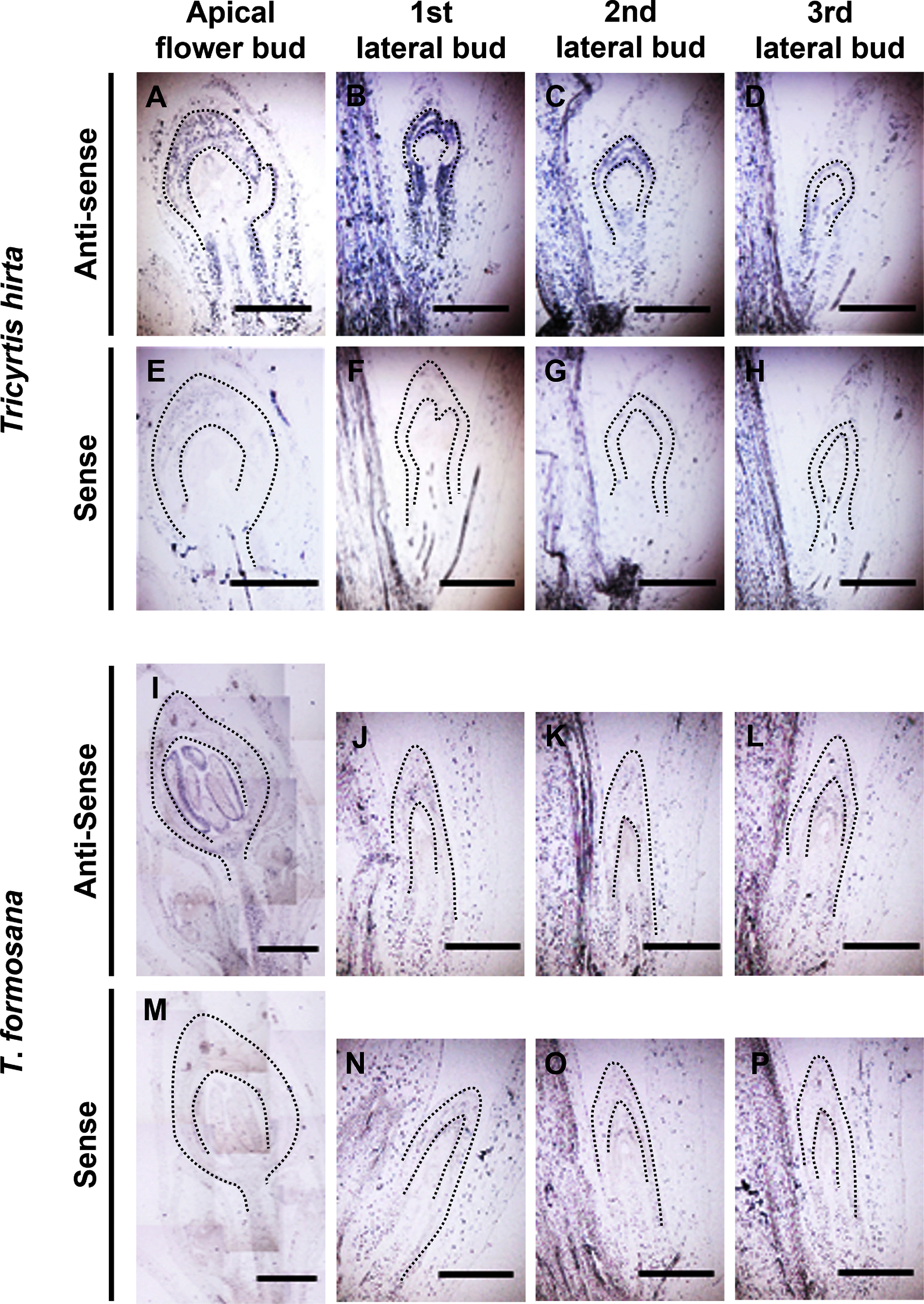
Figure 5. *In situ* hybridization of *ThirLFY* and *TforLFY* in apical flower buds and lateral buds of *Tricyrtis hirta* (A–H) and *T*. *formosana* (I–P). (A, E, I, M) Apical flower buds. (B, F, J, N) First lateral buds below the apical flower buds. (C, G, K, O) Second lateral buds below the apical flower buds. (D, H, L, P) Third lateral buds below the apical flower buds. Scale bars=1 mm.

In order to elucidate the expression patterns of *ThirLFY* and *TforLFY*, RT-PCR and *in situ* hybridization analyses were performed. RT-PCR analysis revealed that both *ThirLFY* and *TforLFY* were detected in apical flower buds, but not in roots, stems and leaves (Figure 4). When apical flower buds of potted plants had grown to ca. 5 mm in length, *in situ* hybridization analysis was performed using apical and lateral buds (Figure 5). *ThirLFY* transcripts were detected in both apical and lateral buds in *T*. *hirta*. On the other hand, *TforLFY* transcripts were detected only in apical buds but not in any lateral buds of *T*. *formosana*. No signals were detected with the sense probe as negative control in all samples.

## Discussion

In the present study, we have isolated *LFY*-homologous genes from two *Tricyrtis* spp., *T*. *hirta* and *T*. *formosana*, showing different types of inflorescence architecture. Deduced amino acid sequences of *ThirLFY* and *TforLFY* showed high homologies to those of *LFY*-homologous genes of other plant species and shared well-conserved N- and C-terminal regions (Figure 1A, B). Furthermore, overexpression of *ThirLFY* or *TforLFY* induced early flowering and development of secondary inflorescences (Figure 3), which are typical phenotypes of transgenic *A. thaliana* plants overexpressing *LFY* (Weigel and Nilsson 1995). These results indicate that *ThirLFY* and *TforLFY* function as *LFY* in *T*. *hirta* and *T*. *formosana*, respectively.

In the genus *Cornus* (Cornaceae), some amino acid substitutions were found in C-terminal region containing an essential domain for DNA-binding specificity among deduced amino acid sequences of *LFY*-homologous genes of six species showing different types of inflorescence architecture, suggesting that inflorescence architecture could be determined by different function of *LFY*-homologous genes (Liu J et al. 2013). On the other hand, in the present study, only three amino acid substitutions were found in N-terminal regions between ThirLFY and TforLFY: Leu/Ser at position 25, Pro/Ala at position 40, and Phe/Tyr at position 95 (Figure 1A, Supplementary Figure S1). In particular, at position 95, ThirLFY conserves hydrophobic residues as in the case of most LFYs from other plant species, whereas TforLFY conserves hydrophilic residues (Supplementary Figure S1). In LFY, N-terminal regions include some alpha helix structures and short leucine zippers called SAMs, which play an essential role in precise conformation and homodimerization (Sayou et al. 2016; Siriwardana and Lamb 2012). Therefore, it is possible that ThirLFY and TforLFY have different functions in floral development and inflorescence architecture determination in *Tricyrtis* spp. However, no apparent morphological difference was observed between transgenic *A*. *thaliana* plants overexpressing *ThirLFY* and those overexpressing *TforLFY* (Figure 3), suggesting that *ThirLFY* and *TforLFY* may have no functional difference.

Different expression patterns of *LFY*-homologous genes have been reported to resulted in different inflorescence architectures in many plant species (Blazquez et al. 1997; Kyozuka et al. 1998; Souer et al. 1998). In the present study, *ThirLFY* expression was detected in both apical and lateral buds in *T*. *hirta* which produces both apical and lateral flowers, whereas *TforLFY* expression was detected only in apical buds in *T*. *formosana* which produces only apical flowers (Figures 4, 5). Thus, different expression patterns of *LFY*-homologous genes are consistent with different flower production patterns among *T*. *hirta* and *T*. *formosana*, and two different types of inflorescence architecture in *Tricyrtis* spp. are likely to be caused by different expression patterns of *LFY*-homologous genes. Promoter analysis for *ThirLFY* and *TforLFY* using GUS reporter gene are now in progress.

In *A*. *thaliana*, *LFY* expression is directly or indirectly regulated by several transcriptional factors, such as FT and TFL1. FT promotes floral transition through activation of floral meristem identity genes such as *LFY*, while TFL1 represses expression of such genes to maintain vegetative state of meristem tissues (Kardailsky et al. 1999; Ratcliffe et al. 1999; Zhu et al. 2020). In addition, *TFL1* have also been reported to be involved in inflorescence architecture determination. In *A*. *thaliana*, *tfl1* mutants have terminal flowers and secondary inflorescences from rosette leaf axils, and such inflorescence architecture is apparently different from wild-type plants (Liljegren et al. 1999; Shannon and Meeks-Wagner 1991). Similar changes in inflorescence architecture have also been observed in *tfl1* mutants of other plant species, such as *Antirrhinum majus*, *Oryza sativa* and *Solanum lycopersicum* (Cremer et al. 2001; Liu C et al. 2013; Pnueli et al. 1998). It is quite possible that, in addition to *LFY*-homologous genes, *FT*- and *TFL1*-homologous genes are also involved in inflorescence architecture determination in *Tricyrtis* spp. Thus, we are now examining isolation and characterization of *FT*- and *TFL1*-homologous genes in both *T*. *hirta* and *T*. *formosana*.

## Abbreviations

CaMV: cauliflower mosaic virus
CTAB: cetyltrimethylammonium bromide
DIG: digoxigenin
FAA: formaldehyde-acetic acid-alcohol
FT: FLOWERING LOCUS T
GUS: β-glucuronidase
HPT: hygromycin phosphotransferase
LFY: LEAFY
NOS: nopaline synthase
NPTII: neomycin phosphotransferase II
PCR: polymerase chain reaction
RACE: rapid amplification of cDNA ends
RT-PCR: reverse-transcription polymerase chain reaction
SAM: sterile alpha motif
TFL1: TERMINAL FLOWER 1

## Description of supplementary files

Supplementary Table S1. List of primers used in the present study.

Supplementary Table S2. List of accession numbers of *LFY*-homologous genes used for phylogenetic analysis.

Supplementary Figure S1. Alignment analysis of N-terminal regions of LFYs based on amino acid sequences.
