# Supplementary material for: Isolation and characterization of LEAFY-homologous genes from two Tricyrtis spp. showing different inflorescence architecture
Source: Plant Biotechnol (Tokyo). 2025 Jun 25;42(2):155–61. doi: 10.5511/plantbiotechnology.25.0225a (PMC12235437; doi:10.5511/plantbiotechnology.25.0225a)

Supplementary Table S1. List of primers used in the present study.

Primer	Sequence	Note
3R-LFY-Fw4	CTTGTTTCATCTCTACGAGCAGTG	For 3'-RACE
3site adapter primer	CTGATCTAGAGGTACCGGATCC	For 3'-RACE
5R-LFY-S1	CTCCGCCAGCTCTGTCACC	For 5'-RACE (1st PCR)
5R-LFY-A1	CAAGCCCCTACGTTCTCTCC	For 5'-RACE (1st PCR)
5R-LFY-S2	TCCTTACGGCTGCTAACGAG	For 5'-RACE (2nd PCR)
5R-LFY-A2	CGTAGCAGTGAACGTAGTGTCG	For 5'-RACE (2nd PCR)
ThirLFY-Fw	GAACCCAGGCCACCAAACC	For amplification of full-length cDNA (*ThirLFY*)
ThirLFY-Rev	AGCCAATAGCTTCTAAACTTAC	For amplification of full-length cDNA (*ThirLFY*)
TforLFY+UTR-Fw	GCAAACTAACAAGTTATGATTGTTGG	For amplification of full-length cDNA (*TforLFY*)
TforLFY+UTR-Rev	GGCCAATAGCTTCTAAACTTACATTG	For amplification of full-length cDNA (*TforLFY*)
TrLFY in fusion-Fw	CACGGGGGACTCTAGAATGGACCCCGG CGACGCCTTC	For cloning ORF into pIG121Hm (*ThirLFY* or *TforLFY*)
TrLFY in fusion-Rev	GATCGGGGAAATTCGAGCTCTCAAAAC ATCGGTGGCGGTGG	For cloning ORF into pIG121Hm (*ThirLFY* or *TforLFY*)
ThirLFY-RT Fw	CGAGGTGTTCCAAGGCTACGG	For RT-PCR analysis (*ThirLFY*)
ThirLFY-RT Rv	GTCGATGTCCCAACCGAGTGG	For RT-PCR analysis (*ThirLFY*)
TforLFY-RT Fw	CCTGTTTCACCTTTACGAGCAG	For RT-PCR analysis (*TforLFY*)
TforLFY-RT Rv	CTCCCAATACTCCAATACCTATAA	For RT-PCR analysis (*TforLFY*)

Supplementary Table S2. List of *LFY*-homologous genes used for phylogenetic analysis.

**Gene**	**Specie**	**Accession No.**
AcOrcLFY	*Anacamptis pyramidalis*	AB088457
AFL1	*Malus domestica*	AB162028
AFL2	*Malus domestica*	AB162034
ALF	*Petunia hybrida*	AF030171
AtLFY	*Arabidopsis thaliana*	NM001345500
BjLFY	*Brassica juncea*	DQ471932
BnLFY	*Brassica napus*	KT715498
BoLFY	*Brassica oleracea*	KT715496
CjNdly	*Cryptomeria japonica*	AB074568
ClLFY	*Chrysanthemum lavandulifolium*	AY559245
CoLFY-1	*Cydonia oblonga*	AB162031
CoLFY-2	*Cydonia oblonga*	AB162037
CrLFY	*Citrus reticulata*	DQ995349
CsLFY	*Citrus sinensis*	AY338976
CuLFY	*Citrus unsiu*	DQ995347
FA	*Solanum lycopersicum*	NM001247459
FLO	*Antirrhinum majus*	M55525
GinLFY	*Ginkgo biloba*	GU563897
GmLFY	*Glycine max*	DQ448809
IacLFY	*Ionopsidium acaule*	AY219226
LcLFY	*Lotus corniculatus*	AY770393
LlLFY	*Lilium longiflorum*	EF458319
OrcLFY	*Ophrys tenthredinifera*	AB088445
OSL	*Oryza sativa*	AF065992
PaLFY	*Picea abies*	AY701763
PcLFY	*Pinus caribaea*	AY640316
PpLFY-1	*Pyrus pyrifolia*	AB162029
PpLFY-2	*Pyrus pyrifolia*	AB162035
PrLFY	*Pinus radiata*	AF109149
RcLFY	*Rosa chinensis*	MN119279

(Continued)

Gene	Specie	Accession No.
TaLFY	*Triticum aestivum*	OR753911
TforLFY	*Tricyrtis formosana*	AB829896
ThirLFY	*Tricyrtis hirta*	AB829895
ZmLFY	*Zea mays*	DQ343237

Supplementary Figure S1. Alignment analysis of N-terminal regions of LFYs based on amino acid sequences. Red arrowheads indicate amino acid substitutions between ThirLFY and TforLFY. Red and blue boxes indicate hydrophobic and hydrophilic amino acid residues at position 95, respectively.

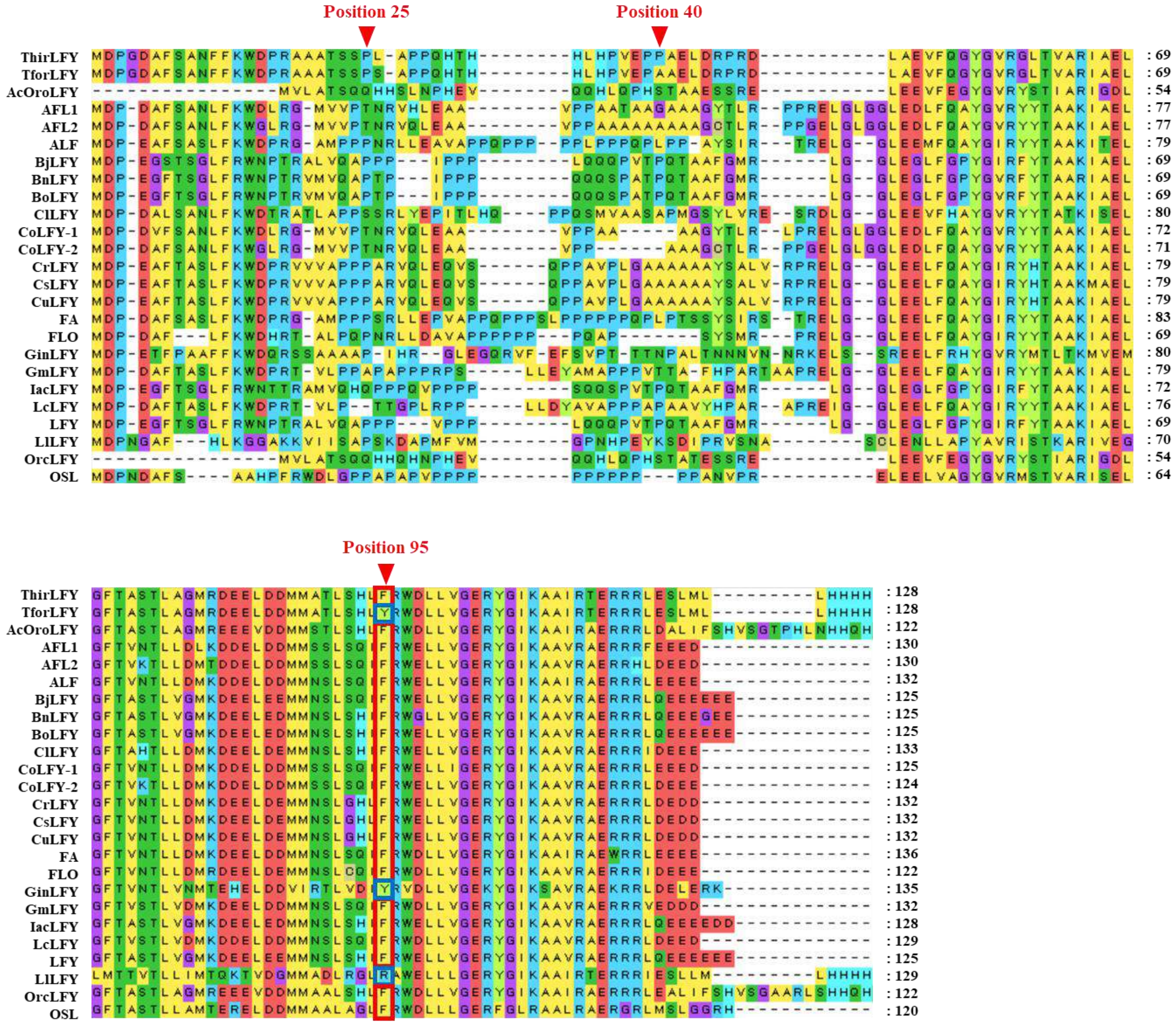

Supplement: Supplementary Data [file plantbiotechnology-42-2-25.0225a-s001.pdf]
